# Comparison of Radiographic Progression-Free Survival and PSA Response on Sequential Treatment Using Abiraterone and Enzalutamide for Newly Diagnosed Castration-Resistant Prostate Cancer: A Propensity Score Matched Analysis from Multicenter Cohort

**DOI:** 10.3390/jcm8081251

**Published:** 2019-08-19

**Authors:** Kazumasa Komura, Yuya Fujiwara, Taizo Uchimoto, Kenkichi Saito, Naoki Tanda, Tomohisa Matsunaga, Atsushi Ichihashi, Takeshi Tsutsumi, Takuya Tsujino, Yuki Yoshikawa, Yudai Nishimoto, Tomoaki Takai, Koichiro Minami, Kohei Taniguchi, Tomohito Tanaka, Hirofumi Uehara, Hajime Hirano, Hayahito Nomi, Naokazu Ibuki, Kiyoshi Takahara, Teruo Inamoto, Haruhito Azuma

**Affiliations:** 1 Department of Urology, Osaka Medical College, Osaka 569-8686, Japan; 2 Translational Research Program, Osaka Medical College, Osaka 569-8686, Japan; 3 Department of Urology, Saiseikai-Nakatsu Hospital, Osaka 530-0012, Japan; 4 Department of Urology, Osaka Medical College Mishima-Minami Hospital, Osaka 569-0856, Japan; 5 Department of Urology, Aijinkai-Takatsuki Hospital, Osaka 569-1192, Japan; 6 Division of Urology, Department of Surgery, Brigham and Women’s Hospital, Boston, MA 02115, USA; 7 Department of Urology, Hirakata Municipal Hospital, Osaka 573-1013, Japan; 8 Department of Urology, Fujita-Health University School of Medicine, Nagoya 470-1192, Japan

**Keywords:** castration-resistant prostate cancer, abiraterone, enzalutamide, propensity score matched analysis, radiographic progression-free survival, sequential treatment

## Abstract

*Background*: There is emerging evidence that radiographic progression-free survival (rPFS) is highly correlated with overall survival (OS), potentially serving as an indicator of treatment outcome for castration-resistant prostate cancer (CRPC). The objective of this study is to assess rPFS and prostate specific antigen (PSA) response in sequential treatment using androgen signaling inhibitors (ASIs) including abiraterone and enzalutamide in newly diagnosed CRPC. *Methods*: Propensity score matching was performed to reduce bias by confounding factors between first-line ASIs. The primary endpoints of the study included rPFS, time to PSA progression (TTPP), and PSA response. *Results*: A paired-matched group of 184 patients were identified. From the initiation of first-line ASIs, there was no significant difference in rPFS, TTPP, and PSA response between treatment arms. From the initiation of second-line ASIs, enzalutamide following abiraterone consistently exhibited longer rPFS (median: 7 and 15 months, *p* = 0.04), TTPP, and better PSA response compared to the reverse, whereas OS did not reach significance (median: 14 and 23 months, *p* = 0.35). *Conclusion*: Although the effect of ASIs as the first line was similar, the extent of cross-resistance might differ towards less resistance in enzalutamide following abiraterone than the reverse.

## 1. Introduction

Prostate cancer is one of the most common malignancies in men [1]. Although androgen deprivation therapy (ADT) offers certain remissions lasting 1 to 2 years in most patients, cancer cells eventually develop castration-resistant prostate cancer (CRPC) through multiple mechanisms [2,3,4,5]. Over the past decade, several new agents have been approved for the treatment of CRPC including two androgen signaling inhibitors (ASIs), namely, abiraterone acetate plus prednisone (referred to from here on as ‘abiraterone’) and enzalutamide. Following the first approval of these ASIs for the treatment of CRPC previously treated with docetaxel [6,7], these agents have been further approved in chemotherapy-naïve settings [8,9], even with the initiation of ADT [10,11]. As of now, these ASIs have been widely used due to their durable efficacy, convenient oral administration, and favorable toxicity profiles in real-world practice [12,13].

There have been a myriad of retrospective studies reporting the sequential use of ASIs for the treatment of CRPC—namely ‘abiraterone following enzalutamide’ and ‘enzalutamide following abiraterone’ [14,15,16,17,18,19,20,21,22,23,24,25,26]. The data from these results seems to indicate cross-resistance between these ASIs. In particular, a modest response of abiraterone after progression on docetaxel and enzalutamide was observed in patients after discontinuation from the AFFIRM trial [7], in which less than 10% of patients achieved a ≥50% decline of prostate specific antigen (PSA) with subsequent abiraterone [18,21]. In 2017, the result of a phase 4, single-arm study (ClinicalTrials. Gov, NCT02116582) of enzalutamide in patients who had progressive disease following prior abiraterone treatment was reported from a multi-institutional collaboration in Europe [27]. This revealed that, despite the cross-resistance between these ASIs, enzalutamide following abiraterone appears to be active in CRPC with or without prior chemotherapy. In addition, most recently, the results from a phase 2 randomized cross-over trial (ClinicalTrials. Gov, NCT02125357) using these ASIs [28] exhibited that PSA response and time to PSA progression (TTPP) on second-line ASIs were significantly associated with favorable outcomes in patients treated with enzalutamide following abiraterone.

Several post-hoc studies from the results of randomized control trials (RCTs) revealed emerging evidence that radiographic progression-free survival (rPFS) is highly correlated with overall survival (OS), potentially serving as an indicator of treatment outcome in CRPC patients [29,30,31]. In 2015, Morris et al. first reported that the Spearman’s correlation coefficient (SCC) between rPFS and OS was 0.71 (95%CI: 0.65–0.77) in pot-hoc analysis from COU-AA-302 study (abiraterone vs placebo for chemotherapy-naïve metastatic CRPC) [30]. Subsequently, Rathkopf et al. showed similar SCC between rPFS and OS (SCC: 0.72, 95%CI: 0.67–0.76) from the PREVAIL study (enzalutamide vs placebo for chemotherapy-naïve metastatic CRPC) [29]. Recently, the results from a prospective multicenter observational cohort study in 406 metastatic CRPC patients treated with ASIs or docetaxel as a first line (NCT03075735) demonstrated Pearson’s correlation with OS of 0.65 in rPFS and 0.54 in TTPP [31]. In the present study, we conducted propensity score-matched analysis for the treatment of newly diagnosed CRPC patients treated with sequential ASIs from the first line, and assessed rPFS as well as PSA response in each line to investigate the extent of cross-resistance with these ASIs and the impacts on their prognosis.

## 2. Materials and Methods

This multicenter cohort study was designated to assess the treatment outcome of sequential therapy using abiraterone acetate (1000 mg) plus prednisone (5 mg twice daily) and enzalutamide (160 mg) in patients with newly diagnosed CRPC. The study design was approved by the institutional review board (IRB approval number: RIN-750-2571) and performed in accordance with the ethical standards of the World Medical Association Declaration of Helsinki [32]. CRPC was diagnosed by a serum testosterone level of <50 ng/dL, and either PSA progression (an increase of 25% and an absolute increase of 2 ng/mL or more above the PSA nadir) or radiographic progression (defined by PCWG2 guidelines) [33]. According to the previous reports showing a marginal survival benefit for CRPC remaining on LHRH analogs during subsequent therapies [34,35], all the patients indefinitely underwent LHRH analogs using ASIs with the treatment. In the present study, patients treated with ASIs for hormone-naïve prostate cancer were excluded.

**Figure 1:**
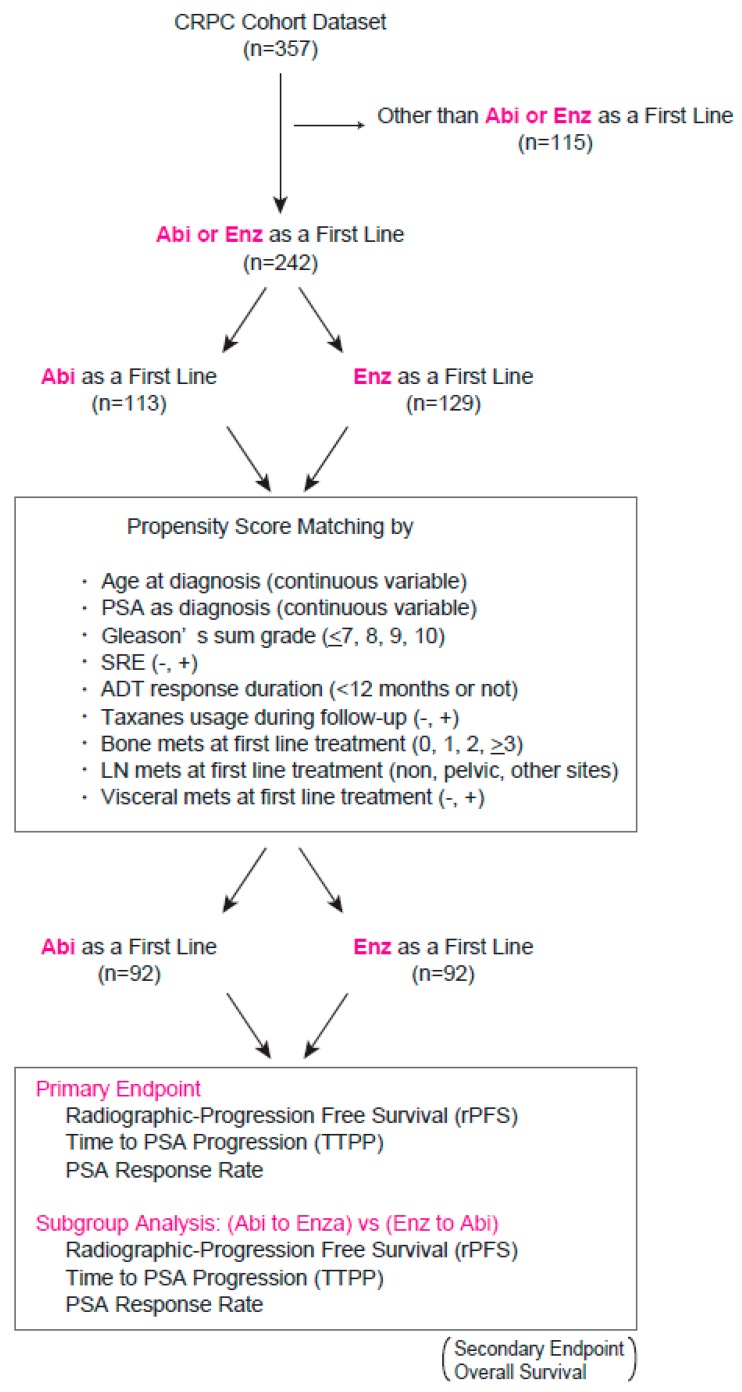
Study design and inclusion criteria of the propensity score-matched analysis in patients newly diagnosed with castration-resistant prostate cancer (CRPC). A 1:1 matching across the two treatment arms was performed using the nearest neighbor method with a 0.2-width caliper of the standard deviation of the logit of the propensity scores.

Figure 1 represents a scheme of the study in which a total of 357 CRPC patient records were collected, followed by a logistic regression propensity score model stratified by the type of first-line ASIs including abiraterone and enzalutamide. To estimate the propensity score (conditional probability), the following variables were included in the regression model: age at diagnosis (continuous variable), PSA at diagnosis (continuous variable), Gleason’s sum grade (<7, 8, 9, 10), skeletal-related events (SRE) during follow-up (−, +), ADT response duration (<12 months or not), taxane usage during follow-up (−, +), bone metastasis at first-line treatment (0, 1, 2, >3), LN metastasis at first-line treatment (none, pelvic, other sites), and visceral metastasis at first-line treatment (−, +). Missing values were included in the model as separate categories as missing data may systematically differ between the two treatment groups. A 1:1 matching (without replacement) across the two treatment groups was achieved by the nearest neighbor method with a 0.2-width caliper of the standard deviation of the logit of the propensity scores to reduce bias by those potential confounding factors. Matching was carried out using the JMP Pro Add-In package version 13.0.0 (SAS Institute Inc., Cary, NC, USA).

The primary endpoints of the study included rPFS, time to PSA progression (TTPP), and PSA response. The secondary endpoint involved overall survival (OS). Radiographic progression was evaluated based on PCWG2 guidelines [33]. As specified, development or progression of lymph nodes greater than 2 cm by spiral computed tomography (CT), as well as any other visceral or soft tissue lesions, were measured according to RECIST guidelines (ver1.1) [36]. For bone metastasis, radiological progression was defined as when at least two or more new lesions are seen on a bone scan compared with a prior scan. In cases where the scan findings are ambiguous (i.e., suggestive of a flare reaction or a trauma), confirmation by other imaging modalities such as MRI, fine-cut CT, and positron emission tomography (PET/CT) were required, which is not necessary if multiple new areas of uptake are observed [37]. Serum PSA level was measured every month from the baseline assessment at the initiation of the first-line treatment. PSA response was defined as nadir level in 6 months divided by the baseline PSA level. PSA progression, also referred to as TTPP, was also defined based on the PCWG2 guideline [33]. Briefly, an increase of 25% and an absolute increase of 2 ng/mL or more above the PSA nadir was considered PSA progression.

Clinical stages in each patient were evaluated by magnetic resonance imaging (MRI), computed tomography (CT), ultrasound, and chest-X rays, and other patient information including performance status (Eastern Cooperative Oncology Group, ECOG-PS) and all the clinical laboratory measurement in peripheral blood (CRP, albumin, alkaline phosphatase, lactate dehydrogenase, neutrophils, lymphocytes, platelets, and hemoglobin) were recorded at an initiation of first-line treatment. Follow-up CT to detect any findings suspected of disease progression were scheduled every 3 months from the diagnosis of CRPC. Based on the PCWG2 guidelines [33], MRI, bone scintigraphy, and PET/CT were further performed when necessary for a definitive diagnosis of disease progression. Follow-up was calculated from the day of initiation of first-line treatment to the day of death or final visit.

Clinicopathological findings in the analysis included patient age, PSA value, skeletal-related events (SRE), ECOG-PS, Gleason’s sum score, and site of metastasis. The distribution of each factor was assessed by a contingency table with a Chi-square analysis. Kolmogorov–Smirnov normality was examined to check normal distribution in continuous variables, followed by a Student’s *t*-test or one-way ANOVA to assess the difference between the variables. For variables with non-normal distribution, a Wilcoxon or Kruskal–Wallis test was performed to assess the difference. A Kaplan–Meier analysis was carried out to estimate the survival free ratio, and a log-rank test was performed to compare the differences between assigned patient groups. On univariate and multivariate analysis, Cox proportional-hazard regression models were used to estimate crude hazard ratios (HR) followed by calculating covariate-adjusted HR. All statistical tests were two-sided, with *p* < 0.05 considered to indicate statistical significance. All analyses were performed using JMP Pro version 13.0.0 (SAS Institute Inc., Cary, NC, USA).

## 3. Results

**Figure 2:**
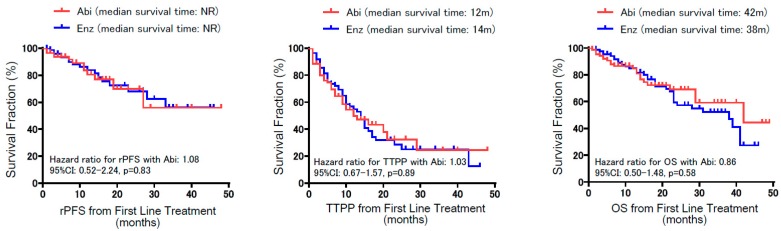
Kaplan–Meier curves for radiographic progression-free survival (rPFS), time to prostate specific antigen (PSA) progression (TTPP), and overall survival (OS) from the initiation of the first-line treatment. Note that there was no significant difference in rPFS, TTPP, and OS between abiraterone and enzalutamide from the first-line treatment.

Figure 1 represents the study design for comparing the treatment outcome of abiraterone and enzalutamide from the first-line treatment. Of all 357 CRPC patients in the cohort, 242 patients were treated with either abiraterone (113 patients) or enzalutamide (129 patients) as the first-line treatment. To reduce any bias due to potential confounders that could affect treatment outcome between both arms, propensity score matching was performed using putative variables, as shown in Figure 1, from which 92 patients in each arm were identified as pair-matched groups. Table 1 exhibits that all the variables in the pair-matched groups had no significant differences between treatment arms. In total, 55 (29.9%) patients deceased during their follow-up, and the median follow-up time for patients living and dead were 17 and 13 months, respectively. Of note, the matched cohort included 57 of M0 CRPC patients at the initiation of the first-line treatment. Two-year OS rates in M0 and M1 CRPC patients from initiation of the first-line treatment were 74.3% and 55.7%, respectively (*p* = 0.03). Figure 2 illustrates rPFS, TTPP, and OS from the time of initiation of the first line according to the treatment. Kaplan–Meier curves showed no significant difference in rPFS, TTPP, and OS between abiraterone and enzalutamide from the first-line treatment. The median time to treatment failure was 12 months in abiraterone and 15 months in enzalutamide, with no significant difference between the arms (*p* = 0.30).

**Figure 3:**
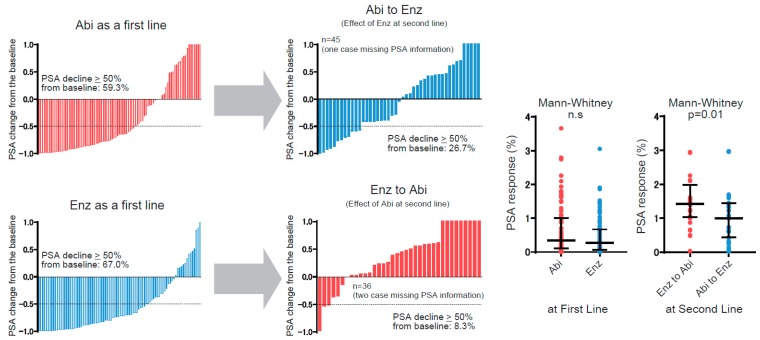
Waterfall plot illustrating the PSA response to first and second line androgen signaling inhibitors (ASIs). Dotted lines express the level of >50% PSA decline. In the first-line treatment, PSA decline of more than 50% from the baseline is observed in 59.3% (54 of 92) for abiraterone and 67% (60 of 92) for enzalutamide, with no significant difference (Chi-square: *p* = 0.28). In the second-line treatment, PSA decline of more than 50% from the initiation of second-line treatment is significantly less observed in abiraterone as a second line (8.3%) compared with enzalutamide following abiraterone (26.7%) (Chi-square: *p* = 0.03). PSA response from the baseline at each line is shown in the right panel.

**Figure 4:**
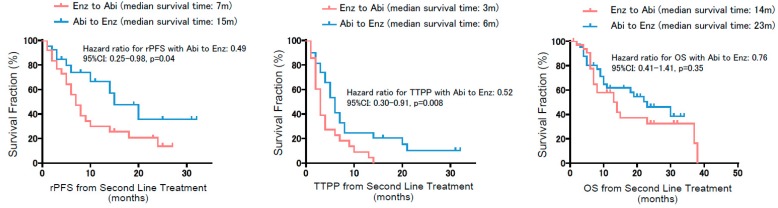
Kaplan–Meier curves for radiographic progression-free survival (rPFS), time to PSA progression (TTPP), and overall survival (OS) from the initiation of the second-line treatment. Note that rPFS and TTPP from the initiation of the second line significantly favored enzalutamide following abiraterone compared to vice versa.

To further examine the effect of ASIs in sequential usage, we conducted subgroup analysis for patients who were treated with second-line ASIs—namely, either abiraterone following enzalutamide, or vice versa. There were 84 out of 184 patients who had subsequent second-line ASIs, whereas 20 patients were treated with taxanes as a second line. Comparing clinical characteristics between ‘abiraterone to enzalutamide in 46 patients’ and ‘enzalutamide to abiraterone in 38 patients’ showed similar patient backgrounds except for the PSA decline of more than 50% with the first-line ASIs (Table 2). In short, patients treated with ‘enzalutamide following abiraterone’ were less likely to achieve a PSA decline of ≥50% from the baseline at their first-line treatment compared to ‘abiraterone following enzalutamide’ (*p* = 0.037). With regard to PSA kinetics throughout the sequential treatment using these ASIs, we separately assessed PSA response in the first and second line (Figure 3). In the first-line treatment, PSA decline of more than 50% from the baseline was observed in 59.3% (54 of 92) for abiraterone and 67% (60 of 92) for enzalutamide, with no significant difference (Chi-square: *p* = 0.28). A Mann–Whitney test to assess PSA response between ASIs as the first line also exhibited no significant difference. To interrogate cross-resistance across these ASIs, we next investigated PSA response on second-line ASIs (i.e., the effect of enzalutamide following abiraterone and vice versa). Waterfall plots exhibited that PSA decline of more than 50% from the initiation of second-line treatment was significantly less observed in abiraterone as a second line (8.3%) compared to enzalutamide following abiraterone (26.7%) (*p* = 0.03). PSA response between these ASIs as the second line also exhibited better PSA response in enzalutamide following abiraterone than for the reverse (*p* = 0.01). TTPP from the initiation of the second line illustrated a significantly shorter TTPP in abiraterone as a second line (median: 3 months) compared to enzalutamide (median: 6 months), consistently indicating an attenuated effect on PSA in abiraterone as a second line after enzalutamide compared with the reverse (HR: 0.52, 95%PI: 0.30–0.91, *p* = 0.008) (Figure 4). Most importantly, rPFS from the initiation of the second line revealed that enzalutamide following abiraterone was significantly associated with a longer rPFS (median of 15 months) compared to abiraterone following enzalutamide (median of 7 months) (HR: 0.49, 95%CI: 0.25–0.98, *p* = 0.04). Median OS from the initiation of the second line was 14 months in patients with ‘abiraterone following enzalutamide’, and 23 months with ‘enzalutamide following abiraterone’, which did not achieve a significant difference (HR: 0.76, 95%CI: 0.41–1.41, *p* = 0.35).

Given that TTPP and rPFS for the treatment using abiraterone and enzalutamide from the time point of the first line appeared to be similar, whereas these drugs might offer distinct effects when applied a second line, we hypothesized that the extent of cross-resistance for these ASIs differs according to the first line. To assess the clinical impact of the order of sequential ASIs from the initiation of the second line, we performed multivariate analysis including putative variables for the prediction of TTPP and rPFS (Table 3). For the TTPP at the second line, there were several variables serving as an independent predictor of TTPP, including visceral metastasis at the initiation of second line (HR: 3.647, 95%CI: 1.003–23.634, *p* = 0.049), a PSA decline of >50% during first-line treatment (HR: 0.641, 95%CI: 0.401–0.933, *p* = 0.038), and ECOG-PS (HR: 2.154, 95%CI: 1.163–4.154, *p* = 0.014), as well as the treatment sequence (HR: 1.791, 95%CI: 1.091–3.163, *p* = 0.043 for ‘enzalutamide to abiraterone’). For rPFS, visceral metastasis only remained an independent predictor for rPFS (HR: 3.647, 95%CI: 1.182–19.278, *p* = 0.032). Finally, we assessed the correlation of rPFS and TTPP with OS from the initiation of second-line treatment (Table 4). The association with OS appeared robust in rPFS compared to TTPP (Spearman’s correlation coefficients of 0.610 and 0.468, respectively).

## 4. Discussion

In the present study, we investigated rPFS and PSA response in sequential treatment using ASIs for patients newly diagnosed with CRPC. Reducing the effect of confounding variables between treatment arms by propensity matching identified a pair-matched cohort of 184 patients with no significant differences among all variables between treatment arms, which allowed us to compare the treatment outcome for these ASIs with a minimally biased retrospective setting. Since recent studies have suggested that rPFS serves as a robust surrogate in the prediction of OS for treatment using abiraterone [30] and enzalutamide [29], we chose rPFS for both the first and subsequent second line as a primary endpoint as well as PSA response.

There have been a number of retrospective studies assessing sequential treatment using ASIs (i.e., ‘abiraterone following enzalutamide’ [18,19,20,21,23,25,26] and ‘enzalutamide following abiraterone’ [14,15,16,17,20,22,23,24,25,26]). In terms of PSA response, the results from those studies seemed to favor ‘enzalutamide following abiraterone’ [38], whereas no report has demonstrated an improved OS in this treatment sequence. A phase 4 multi-institutional, single-arm, open-label study reported by de Bono et al. enrolled 214 CRPC patients with progressions after ≥24 weeks of abiraterone treatment, and rPFS for the subsequent enzalutamide was examined as the primary endpoint [27]. The median duration of enzalutamide treatment for the second line was 5.7 months, and the median rPFS was 8.1 months. In their study, 69 out of 214 (32.2%) deceased during follow-up, and 69 (32.2%) patients were previously treated with chemotherapy before enzalutamide. In the present study, 55 out of 184 (29.9%) patients deceased during follow-up, and the median rPFS from the initiation of the second line for ‘enzalutamide following abiraterone’ was 15 months, which appeared to be longer than their study [27]. We assume this could be at least in large part due to the fact that the present study included M0 patients at the initiation of the second line (19%), and all the patients were chemotherapy-naïve.

In regard to the PSA response, the phase 2 randomized cross-over trial exhibited that a PSA decline of ≥50% for first-line treatment was 53% in abiraterone versus 73% in enzalutamide (*p* = 0.004), whereas median TTPP was similar (7.4 months in abiraterone vs 8.0 months in enzalutamide: HR = 0.88, 95%CI: 0.61–1.27) [39]. Although there was a similar trend of better PSA response in enzalutamide for the first line, the present study did not show a significant difference in either the achievement of PSA decline ≥50% or PSA response between these ASIs as for the first line (Figure 3). TTPP for the first line also showed no difference between treatment arms. Recently, updated results from a phase 2 study were reported by Khalaf et al. [28]. They showed that PSA response rate and TTPP were better for second-line enzalutamide following abiraterone compared with the reverse, and treatment arm was an independent predictor of PSA progression during second-line treatment. Consistent with their results, the present study also exhibited better PSA response and TTPP in patients treated with enzalutamide as a second line following abiraterone. These data indicate that, although the effect as a first line is similar between these ASIs, the extent of cross-resistance might differ towards less resistance to enzalutamide following abiraterone. The results showed no difference in rPFS and TTPP between ASIs as the first-line treatment, whereas rPFS and TTPP from the initiation of the second line were significantly longer in enzalutamide following abiraterone. Furthermore, we examined an association between rPFS/TTPP and OS from the initiation of second-line ASIs, as shown in Table 4. As expected, both rPFS and TTPP were significantly correlated with OS (*p* < 0.001 in both rPFS and TTPP). Importantly, higher SCC in rPFS was observed compared to that in TTPP (0.601 in rPFS vs 0.468 in TTPP), indicating rPFS is a robust surrogate for predicting OS, which is consistent with the previous report by Lorente et al. [31].

The limitations in the present study involved its retrospective design and relatively small sample size, as well as a lack of consideration of biomarkers such as ARV7 [40]. In addition, the findings in the current study were still subject to selection bias, which we sought to address by using a propensity score-matching model to approximate random assignment. Residual unmeasured confounding factors may have affected the clinical impact of ASIs observed in the study. Finally, multivariate analysis in the current study did not demonstrate treatment sequence and ECOG-PS as independent predictors for predicting rPFS from the initiation of the second-line treatment, which could be due to the low statistical power. To clarify the best treatment strategy for individual patients, further prospective work is warranted.

## 5. Conclusions

We compared rPFS and PSA response in sequential treatment using abiraterone and enzalutamide for newly diagnosed CRPC patients in a retrospective paired-matched cohort by propensity score matching. For the first line, rPFS and TTPP were both similar between ASIs. For the second line, enzalutamide following abiraterone was likely to achieve longer rPFS and TTPP compared to abiraterone following enzalutamide. These findings could allow physicians to select the optimal treatment sequence using ASIs in daily practice.

## Figures and Tables

**Table 1:** Clinical characteristics in 184 CRPC patients adjusted by propensity score matching.

Variables	Total (*n* = 184)	Abi (*n* = 92)	Enz (*n* = 92)	*p* Value
Age (mean ± SD)	73.5 + 7.8	74.0 + 8.0	73.0 + 7.6	0.355
SRE during follow-up				
No (%)	144 (78.3)	72 (78.3)	72 (78.3)	
Yes (%)	40 (21.7)	20 (21.7)	20 (21.7)	1.000
Taxanes during follow-up				
No (%)	159 (86.4)	80 (87.0)	79 (85.9)	
Yes (%)	25 (13.6)	12 (13.0)	13 (14.1)	0.809
ADT response duration				
≥12months (%)	136 (73.9)	65 (70.6)	71 (77.2)	
<12 months (%)	48 (26.1)	27 (29.4)	21 (22.8)	0.809
Median PSA level at diagnosis (ng/mL) (quartile)	124.0 (29.3, 395.6)	124.3 (41.9, 327.1)	93.8 (25.7, 574.8)	0.685
Median PSA level at first line treatment (ng/mL) (quartile)	6.8 (2.3, 30.1)	6.8 (2.0, 30.4)	6.8 (2.5, 30.1)	0.918
Gleason sum score (%)				
≤7	20 (10.9)	9 (9.8)	11 (12.0)	
8	41 (22.3)	20 (21.7)	21 (22.8)	
9	113 (61.4)	56 (60.9)	57 (62.0)	
10	10 (5.4)	7 (7.6)	3 (3.3)	0.598
Local treatment prior to ADT (%)				
Non	156 (84.8)	77 (83.7)	79 (85.9)	
Prostatectomy	16 (8.7)	9 (9.8)	7 (7.6)	
Radiation	8 (4.3)	5 (5.4)	3 (3.3)	
Others	4 (2.2)	1 (1.1)	3 (3.3)	0.311
Initial ADT (%)				
LHRH analog + NAs	162 (88.0)	78 (84.8)	84 (91.3)	
LHRH analog	11 (6.0)	7 (7.6)	4 (4.3)	
NAs	7 (3.8)	5 (5.4)	2 (2.2)	
Others	4 (2.2)	2 (2.2)	2 (2.2)	0.113
Mets at first line treatment (%)				
M0	57 (31.0)	27 (29.4)	30 (32.6)	
M1	127 (69.0)	65 (70.7)	62 (67.4)	0.345
Visceral mets at first line treatment (%)				
No	165 (89.7)	82 (89.1)	83 (90.2)	
Yes	19 (10.3)	10 (10.9)	9 (9.8)	0.809
LN mets at first line treatment (%)				
Non	117 (63.6)	55 (59.8)	62 (67.4)	
Regional	45 (24.5)	23 (25.0)	22 (23.9)	
Non-regional	22 (12.0)	14 (15.2)	8 (8.7)	0.350
No. of bone mets at first line treatment (%)				
0	83 (45.1)	40 (43.5)	43 (46.7)	
1	23 (12.5)	11 (12.0)	12 (13.0)	
2	15 (8.2)	8 (8.7)	7 (7.6)	
>3	63 (34.2)	33 (35.9)	30 (32.6)	0.948
ECOG-PS (%)				
0	104 (56.5)	51 (55.4)	53 (57.6)	
1	66 (35.9)	35 (38.0)	31 (33.7)	
≥2	14 (7.6)	6 (6.6)	8 (8.7)	0.684
Neutrophil-lymphocyte ratio at first line treatment (mean ± SD)	2.99 ± 2.49	3.08 ± 2.93	2.84 ± 1.59	0.648
Hb at first line treatment (g/dL) (mean ± SD)	12.2 ± 1.8	12.1 ± 1.8	12.3 ± 1.8	0.627
Platelet count at first line treatment (10^3^/uL) (mean ± SD)	213 ± 75	215 ± 80	210 ± 71	0.640
ALP at first line treatment (U/L) (quartile)	248 (202, 350)	252 (199, 370)	246 (213, 344)	0.884
LDH at first line treatment (U/L) (quartile)	200 (182, 238)	201 (177, 231)	200 (185, 240)	0.675
Albumin (g/dL) (quartile)	4.1 (3.8, 4.4)	4.1 (3.6, 4.3)	4.2 (3.9, 4.4)	0.126
CRP (mg/dL) (quartile)	0.1 (0.05, 0.32)	0.1 (0.05, 0.23)	0.1 (0.05, 0.48)	0.670

CRPC: castration-resistant prostate cancer, Abi: abiraterone, Enz: enzalutamide, SD: standard deviation, SRE: skeletal-related events, PSA: prostate-specific antigen, ADT: androgen deprivation therapy, LN: lymph node, Mets: metastasis, ECOG-PS: Eastern Cooperative Oncology Group performance status, Hb: hemoglobin, ALP: alkaline phosphatase, LDH: lactate dehydrogenase, CRP: C-reactive protein, LHRH: luteinizing hormone-releasing hormone, NAs: nonsteroidal antiandrogens.

**Table 2:** Patient characteristics in 84 CRPC patients treated with ASIs as 2nd treatment.

Variables	Total (*n* = 84)	Abi to Enz (*n* = 46)	Enz to Abi (*n* = 38)	*p* Value
Age (mean ± SD)	73.0 + 7.9	71.8 + 7.3	74.4 + 8.4	ns
SRE during follow-up				
No (%)	63 (75.0)	32 (69.6)	31 (81.6)	
Yes (%)	21 (25.0)	14 (30.4)	7 (18.4)	ns
Median PSA level at 2nd line treatment (ng/mL) (quartile)	21.3 (3.8, 93.2)	21.5 (8.1, 61.3)	21.0 (3.4, 94.3)	ns
Gleason sum score (%)				
≤7	11 (13.1)	8 (17.4)	3 (7.9)	
8	16 (19.0)	6 (13.0)	10 (26.3)	
9	53 (63.1)	30 (65.2)	23 (60.5)	
10	4 (4.8)	2 (4.4)	2 (5.3)	ns
Mets at 2nd line treatment (%)				
M0	16 (19.0)	9 (19.6)	7 (18.4)	
M1	68 (81.0)	37 (80.4)	31 (81.6)	ns
Visceral mets at 2nd line treatment (%)				
No	75 (88.2)	39 (84.8)	36 (94.7)	
Yes	9 (11.8)	7 (15.2)	2 (5.3)	ns
LN mets at 2nd line treatment (%)				
No	59 (70.2)	31 (67.4)	28 (73.7)	
Yes	25 (29.8)	15 (32.6)	10 (26.3)	ns
Bone mets at 2nd line treatment (%)				
No	25 (29.8)	14 (30.4)	11 (29.0)	
Yes	59 (70.2)	32 (69.6)	27 (71.1)	ns
Taxanes during follow-up				
No	72 (85.7)	41 (89.1)	30 (79.0)	
Yes	12 (14.3)	5 (10.9)	8 (21.0)	ns
ECOG-PS (%)				
0	34 (40.5)	17 (37.0)	17 (44.7)	
1	45 (53.6)	27 (58.7)	18 (47.4)	
≥2	5 (5.9)	2 (4.4)	3 (7.9)	ns
PSA decline ≥50% at first line treatment				
No (%)	43 (51.2)	28 (60.9)	15 (39.5)	
Yes (%)	41 (48.8)	18 (39.1)	23 (60.5)	0.037

CRPC: castration-resistant prostate cancer, Abi: abiraterone, Enz: enzalutamide, SD: standard deviation, SRE: skeletal-related events, PSA: prostate-specific antigen, ADT: androgen deprivation therapy, LN: lymph node, Mets: metastasis, ECOG-PS: Eastern Cooperative Oncology Group performance status.

**Table 3:** Multivariate analysis adjusting with putative variables for the prediction of TTPP and rPFS from the initiation of second line treatment.

	TTPP at 2nd Line				Radiographic PFS			
Variables	HR	95%CI		*p* Value	HR	95%CI		*p* Value
Treatment sequence								
Abi to Enz	Ref				Ref			
Enz to Abi	1.791	1.091	3.163	0.043	1.538	0.855	3.019	0.219
Visceral mets at 2nd line treatment								
No								
Yes	3.647	1.003	23.634	0.049	3.647	1.182	19.278	0.032
LN mets at 2nd line treatment								
No	Ref				Ref			
Yes	1.663	0.847	3.406	0.141	1.233	0.784	2.392	0.221
Bone mets at 2nd line treatment								
No	Ref				Ref			
Yes	1.946	0.972	4.071	0.06	1.392	0.872	4.281	0.099
PSA decline ≥50% at first line								
No	Ref				Ref			
Yes	0.641	0.401	0.933	0.038	0.865	0.431	1.283	0.492
ECOG-PS								
0	Ref				Ref			
>1	2.154	1.163	4.154	0.014	1.538	0.699	2.193	0.293

TTPP: time to PSA progression, HR: hazard ratio, CI: confidential interval, ECOG-PS: Eastern Cooperative Oncology Group performance status

**Table 4:** Correlation of rPFS and TTPP with OS from the initiation of second line treatment.

Variables	Spearman’s Correlation Coefficient (SCC) (95%CI)	*p* Value
rPFS	0.601 (0.411–0.722)	<0.001
TTPP	0.468 (0.275–0.625)	<0.001

rPFS: radiographic progression-free survival, TTPP: time to PSA progression, OS: overall survival, CI: confidence interval.

## Abbreviations

rPFS	radiographic progression-free survival
OS	overall survival
CRPC	castration-resistant prostate cancer
ASIs	androgen signaling inhibitors
TTPP	time to PSA progression
ADT	androgen deprivation therapy
RCT	randomized control trial
SCC	Spearman’s correlation coefficient
